# Chemokine receptor CCR6 expression is regulated by miR-518a-5p in colorectal cancer cells

**DOI:** 10.1186/1479-5876-12-48

**Published:** 2014-02-21

**Authors:** Claudia Rubie, Bianca Kruse, Vilma Oliveira Frick, Kathrin Kölsch, Pirus Ghadjar, Mathias Wagner, Friedrich Grässer, Stefan Wagenpfeil, Mathias Glanemann

**Affiliations:** 1 Department of General -, Visceral-, Vascular - and Paediatric Surgery, University of the Saarland, 66421 Homburg/Saar, Germany; 2 Department of Radiation Oncology, Charité University Medicine, Berlin, Germany; 3 Institute of Pathology, University of the Saarland, 66421 Homburg/Saar, Germany; 4 Institute of Virology, University of the Saarland, 66421 Homburg/Saar, Germany; 5 Institute of Medical Biometrics, Epidemiology, and Medical Informatics (IMBEI), University of the Saarland, 66421 Homburg/Saar, Germany

**Keywords:** microRNA, miR-518a-5p, Chemokine receptors, CCR6, CRC

## Abstract

**Background:**

Recently, involvement of the chemokine/receptor system CCL20/CCR6 in colorectal cancer (CRC) progression was shown. Here, we analyzed the functional interaction of miRNA-518-5p (miR-518a-5p) with CCR6 and its impact on CCR6 expression in CRC cells.

**Methods:**

MiR-518a-5p was identified by computer software to potentially interact with CCR6. Hence, functional implications of miR-518a-5p with the 3′UTR of CCR6 were analyzed using the Dual Luciferase Reporter assay system. Confirmation of the predicted target site for miR-518a-5p was achieved by site-directed mutagenesis of the seed sequence in the 3′UTR of CCR6 and subsequent application of the mutated seed sequence in a luciferase assay with miR-518a-5p mimics. Accordingly, two CRC cell lines (Caco-2 and HT-29) were transfected with miR-518a-5p miRNA mimics and gene and protein expression of CCR6 was monitored using qRT PCR and immunocytochemistry, respectively.

**Results:**

Addition of miR-518a-5p led to significant down-regulation of luciferase activity (P < 0.05), which was significantly reversed in a reporter test system containing the mutated seed sequences in the 3′UTR of CCR6. Following transfection of CRC cell lines with miR-518a-5p mimics and subsequent monitoring of CCR6 expression showed significant down-regulation of CCR6 mRNA and CCR6 protein expression in both CRC cell lines under investigation (P < 0.05).

**Conclusions:**

We have shown that miR-518a-5p functionally interacts with CCR6 and that transfection of CRC cells with miR-518a-5p leads to significant CCR6 down-regulation. Consequently, CCR6 expression is regulated by miR-518a-5p in CRC cells indicating that regulation of CCR6 expression by miR-518a-5p might be a regulatory mechanism involved in CRC pathogenesis.

## Background

MicroRNAs (miRNAs) are recently studied evolutionarily conserved, naturally occurring non-coding RNAs which are characterized by their short size (19–25 nucleotides), lack of a poly-A tail and their ability to bind cognate mRNA targets with sequence homology [1,2]. As key control elements of crucial regulatory pathways in plants and animals miRNAs were shown to regulate gene expression post-transcriptionally by binding to the 3′ untranslated regions (UTRs) of target mRNAs, thereby inhibiting mRNA translation [3,4].

To date, miRNAs are known to play a role in a wide range of cellular processes and although a few thousand predicted miRNAs have been identified in a variety of organisms, little is known about their cellular functions. It is estimated that as high as 30% of protein-coding genes could serve as miRNA targets. As miRNAs often regulate multiple transcripts, they are involved in various biological processes comprising cell differentiation, proliferation, apoptosis, metabolism, protein secretion and host-pathogen interactions including viral infection. While several studies have recently described aberrant expression of miRNAs in different cancer entities, it is yet unknown if this directly influences the carcinogenic process [5,6]. Therefore, in cancer cells some miRNAs are described as "tumor suppressor miRNAs" (TSmiRNAs) because they inhibit the translation of proto-oncogenes in normal tissues. In contrast, other miRNAs are referred to as "oncomiRs" because their up-regulation leads to the down-regulation of tumor suppressor genes. As both oncogenes and tumor suppressor genes can be targets of dysregulated miRNAs, the function of a special miRNA may depend highly on the target and the cell environment [7].

In the last decade, also chemokines have been shown to participate in tumor growth and angiogenesis and the lymphatic and even distant spread of malignant tumors [8-13]. The biological effects of chemokines are exerted by interacting with seven-span transmembrane domain receptors coupled to trimeric G proteins, that are selectively found on the surfaces of their target cells. Consequently, chemokines and their receptors my facilitate dissemination of tumor cells at each of the key steps of metastasis like adhering to endothelium, extravasation from blood vessels, angiogenesis, colonization, proliferation and protection from the host response [14]. Moreover, chemokines play an important role in the communication between cancer cells and non-cancerous cells like endothelial cells, neutrophils, fibroblasts and tumor-associated macrophages in the tumor-microenvironment.

Recently, various cancer-related studies demonstrated that specific chemokines and their receptors are dysregulated in CRC and may be involved in the molecular mechanisms controlling CRC pathology. In this respect, interactions between the inflammatory and homeostatic chemokine CCL20 and its receptor CCR6 were shown to be involved in CRC pathology [15,16]. In this context, expression of CCL20/CCR6 was found to be significantly up-regulated in CRC, where the CCL20/CCR6 system was recently shown to be a critical component in the regulation of CRC progression and spread which can also be influenced by chemotherapy [17]. In addition, CCL20 stimulation of CRC cells leads to phosphorylation of an adaptor/scaffolding protein involved in adhesion and migration as well as to increased cancer cell proliferation and migration and the activation of the ERK-MAP kinase and Act pathways [18,19]. The precise mechanisms underlying the regulation of CCL20/CCR6 involvement in CRC remain still unclear. Recently, in CRC the expression of various miRNAs has been demonstrated to be down-regulated [20,21]. Based on the assumption that a down-regulated miRNA may cause an increased expression of its target chemokine, we recently investigated functional interactions of several miRNAs with chemokine ligand CCL20. Accordingly, we have outlined a functional interaction of miR-21 with the 3′UTR of CC– chemokine ligand CCL20 [22]. However, investigating the cellular localization of miR-21 and its target CCL20 revealed that both molecules are expressed predominantly in the microenvironment of CRC tumors [23]. In this study, we aimed to explore functional interactions between potential miRNA candidates and CCL20 receptor CCR6. After identifying miR-518a-5p to potentially interact with CCR6 in a luciferase assay system, we investigated its effect on CCR6 expression in CRC cells transfected with miR-518a-5p.

## Methods

### miRNA targets prediction

The miRNA target sites on the CCR6 (NM_004367.5) 3′UTR were predicted using the following programs: TargetScan Human 5.1, MicroRNA.org, MicroCosm Targets, MiRDB and TargetMiner as presented with the corresponding websites in Table 1.

**Table 1:** miRNA target prediction programs - overview

**Program**	**Website**	**Prediciton hsa-miR-518-5p**
TargetScan Human 5.1	http://www.targetscan.org	**X**
MicroRNA.org	http://www.microrna.org	
MicroCosm Targets	http://www.ebi.ac.uk/enright-srv/microcosm	
MiRDB	http://www.mirdb.org	**X**
TargetMiner	http://www.isical.ac.in/~bioinfo_miu/targetminer20.htm	**X**

### Dual Luciferase Reporter assay

For the luciferase assays dual luciferase reporter vectors were used which contained either the 3′UTR of CCR6 (Luc CCR6 vector), a control with no 3′UTR (Luc No CCR6 vector; both Genecopoeia Inc; Rockville, MD, USA) or a mutated 3′UTR of CCR6 (Luc CCR6 Mut vectors) as presented in Table 2. For the reporter assays 293 T cells were cultured in a 24-well plate for 24 h. At a confluency of approximately 80% cells were cotransfected with Luciferase CCR6 vector, Luciferase No CCR6 vector or Luciferase CCR6 Mut vectors and the miR-518a-5p or negative control Pre-miR (NK) miRNA mimics for 48 hours using Lipofectamine 2000 (Life Technologies, Carlsbad, CA). The activities of Firefly (Photinus pyralis) and Renilla (Renilla reniformis) luciferase were quantified with the Dual luciferase Reporter Assay System from Promega Corp (Madison, WI, USA). For normalisation of transfection differences Firefly luciferase activity was related to Renilla’s counterpart. In general, luciferase activities of luciferase CCR6 vector constructs were normalized to Luciferase No CCR6 vector constructs. All values were normalized to the negative control Pre-miR miRNA Precursors.

**Table 2:** Luciferase assay vectors -overview

**Vector**	**Type**	**Example**
CCR6-3′UTR + luciferase gene (firefly)	Dual luciferase reporter assay system with unmutated regulatory region of CCR6	Luc CCR6 vector (GeneCopoeia) CatalogNo: HmiT002212-MT01
No 3′UTR + luciferase gene (firefly)	Dual luciferase reporter assay without regulatory region of CCR6	Luc No CCR6 vector (GeneCopoeia) CatalogNo: CmiT000001-MT01
Mutated CCR6-3′UTR + luciferase gene (firefly)	Dual luciferase reporter assay with mutated regulatory region of CCR6	Luc CCR6 Mut vectors Lab construction vectors CatalogNo: HmiT002212-MT01 with point mutations in the miR-518a-5p seed sequences
• Mutations in BS1		• Luc CCR6 Mut BS1
• Mutations in BS2		• Luc CCR6 Mut BS2
• Mutations in BS1 + 2		• Luc CCR6 Mut BS1 + 2

### Site-directed mutagenesis

To determine whether the predicted target site for hsa-miR-518a-5p is a functional target site we mutated the seed sequence in the 3′UTR of CCR6 and tested the mutated seed sequence in a subsequent luciferase assay. The predicted target sequence 5′-CTTTGCA-3′ was mutated to 5′-TAGACTC-3′ in seed sequence 1 and 2 of the 3′UTR of CCR6. Site-directed mutations were generated by PCR using the HmiT002212-MT01 reporter vector (GeneCopeia) as a template and the QuikChange II XL site-directed mutagenesis kit (Stratagene, La Jolla, CA) according to the manufacturer’s instructions. In the first step of the mutagenesis process we mutated the first seed sequence (position 222–228) by introducing four point mutations into the CCR6 3′UTR of Luciferase reporter vector HmiT002212-MT01 resulting in the respective mutated vector Luc CCR6 Mut BS1. Likewise, we introduced the same four point mutations into the second seed sequence of the 3′UTR of CCR6 (position 1773–1779) resulting in the respective mutated vector Luc CCR6 Mut BS2. Subsequently, vector Luc CCR6 Mut BS1 was applied as a template to introduce a site-directed mutagenesis into the second seed sequence of the 3′UTR of CCR6. Thus, we have introduced the same four point mutations into the second seed sequence of the 3′UTR of CCR6 of vector Luc CCR6 Mut BS1 resulting in vector Luc CCR6 Mut BS1 + 2 which carries the four point mutations in both seed sequences.

FORWARD: 5′-GTCTCTGATAGGTAGCATTTTCCAGCAATCTGAGAGGAATGTTTTG TAGCTCTAGG-3′ and REVERSE: 5′-CCTAGAGCTACAAAACATTCCTCTCAGATTGCTGGAAAATGCTACC TATCAGAGAC-3′.

The primers used for site-directed mutagenesis of the second seed sequence at position 1773–1779 were:

FORWARD: 5′-CAAAGTCTGTATTTTTAAAGCATGGCTTTGGGTCTGGGAAATAAA AAATGTGTTTTGTACATGAAGTAG-3′ and REVERSE: 5′-CTACTTCATGTACAAAACACATTTTTTATTTCCCAGACCCAAAGCCA TGCTTTAAAAATACAGACTTTG-3′.

The mutagenized plasmids were isolated using the Qiagen Miniprep Kit (Qiagen). The mutations were confirmed by DNA sequencing (Seq-it, Kaiserslautern, Germany) of the region containing the mutation. Large scale plasmid isolations were performed using the GenElute™ HP Plasmid Maxiprep Kit (Sigma Aldrich, Munich, Germany). The modified plasmids were designated Luc CCR6 Mut BS1, Luc CCR6 Mut BS2 and Luc CCR6 Mut BS1 + 2, respectively, as presented in Table 2.

### miRNA assays

miRNA transfection (miR-518a-5p) of HT-29 and Caco-2, cells was performed according to the HiPerFect Transfection Reagent Handbook from Qiagen (Qiagen, Hilden, Germany) and the Dharmacon DharmaFECT RNA transfection protocol (Thermo Fisher Scientific, Waltham, USA). Briefly, cells were trypsinized, counted and on average 5 × 10^5^ cells per well of a 6-well plate, were seeded and overnight incubated under their normal growth conditions. Before transfection of Caco-2 cells 5 nM of miRNA mimics were diluted in 100 μl of DMEM and 6 μl of Dharmafect were diluted in 94 μl of DMEM and incubated for 5 min. Hence the two samples were mixed and incubated for 20 min to allow formation of transfection complexes. Subsequently the samples were incubated for 10 min to allow formation of transfection complexes. Accordingly, the complexes were added drop-wise to the cells and incubated under their normal growth conditions. Gene expression of CCR6 was monitored after 48 and 72 hours after transfection with miRNA miR-518a-5p at the mRNA and at the protein level using qRT PCR and immunocytochemistry, respectively. As a positive control the expression of TWF1 upon transfection of hsa-miR-1 was monitored. Further, control samples with untransfected cells, a mock-transfection with only transfection reagent and a negative control miRNA (NK) were applied. A detailed overview of miRNA control experiments is presented in Table 3.

**Table 3:** miRNA assay control experiments - overview

**Control experiment**	**Type**	**Example**
Positive control miRNA	miRNA that is known to downregulate its target gene	miR-1 Mimic (targeting TWF1)
Negative control miRNA (NK)	A nonsilencing miRNA with no homology to any known mammalian gene	microRNA Mimic negative control (Applied Biosystems)
Mock transfection control	Control experiment where cells go through the transfection process without addition of miRNA	
Untransfected cells control	Control experiment where gene expression analysis is carried out on cells that have not gone through the transfection process	

### Single-strand cDNA synthesis

Isolation of total RNA from cell lines was performed using the Qiagen RNeasy Mini Kit according to manufacturer’s instructions. Integrity of RNA samples was confirmed spectrophotometrically and by electrophoresis on 1% agarose gels.

For cDNA synthesis 1 μg of each cell line total RNA sample was reverse-transcribed in a final reaction volume of 50 μl containing 1× TaqMan RT buffer, 2.5 μM random hexamers, 500 μM each dNTP, 5.5 mM MgCl_2_, 0.4 U/μl RNase inhibitor, and 1.25 U/μl Multiscribe RT. The cycler conditions were 10 min at 25°C, 90 min at 48°C, and 5 min at 95°C.

### Quantitative real-time PCR

The q-RT-PCR for CCR6 mRNA detection was performed using 10 μl 2× Taqman Universal PCR Master Mix II and 1 μl CCR6 gene assay Applied Biosystems Life Technolgies (Carlsbad, CA, USA), 8 μl RNAse-free water and 1 μl cDNA template (20 ng/μl).

The theoretical basis of qRT assays is described in detail elsewhere [24]. Triplicates were run for all reactions together with no template controls and an additional control for DNA contamination where the reverse transcriptase was omitted. As detection system the ABI Prism 7900 sequence detector (Applied Biosystems Life Technologies) was programmed to an initial step of 10 min at 95°C, followed by 40 thermal cycles of 15 s at 95°C and 10 min at 60°C and the log-linear phase of amplification was monitored to obtain C_T_ values for each RNA sample.

The expression level of CCR6 mRNA was analyzed in relation to the levels of the slope matched housekeeping gene Cyclophilin C (CycC) [25]. Conversion of the individual C_T_ values to the linear form was performed according to the 2^-delta C^_T_ method.

### Isolation of total protein

Isolation of protein was performed using frozen tissue or cells in 6-well plates and protein lysates became precipitated by the use of RIPA buffer. Quantification of protein in the samples was conducted using the Pierce BCA Protein Assay Reagent Kit (Pierce, Rockford, IL, USA).

### Immunocytochemistry

Immunocytochemical staining of CRC cells was performed using BD Falcon CultureSlides (BD Falcon Ref.: 354104, BD Biosciences Discovery Labware, Bedford, MA, USA) comprised of a glass slide treated to provide a consistent surface for cell growth and 4 compartmentalized chambers molded from polystyrene. Cells of CRC cell lines HT-29 and Caco-2 were seeded with a density of 1×10^5^ cells per ml on the culture slides. After adhesion cells were transfected with miR-518a-5p (5 μM) according to the Dharmacon DharmaFECT RNA transfection protocol (Thermo Fisher Scientific, Waltham, USA). After an incubation time of 72 hours at 37°C and 5% CO_2_ the supernatant was removed and cells were washed with PBS followed by a 10 min fixation in ice cold methanol. Hence, the chambers were removed followed by sequential washing steps. After blocking of endogenous peroxidase activity with 3% hydrogen peroxide, the sections were treated with avidin and biotin (Avidin/Biotin blocking kit, Vector Laboratories Inc., Burlingame, CA, USA). In addition unspecific binding sites were further blocked for 30 min at room temperature with normal rabbit serum.

Overnight incubation at 4°C with primary goat polyclonal anti-human CRR6 antibody (diluted 1:125, C2099-70B, Biomol, Hamburg, Germany) was followed by incubation of secondary biotinylated rabbit anti-goat IgG antibody and the avidin-biotin-peroxidase reaction (Vectastain ABC ELITE Kit, Vector Laboratories, Burlingame, CA, USA). After colour reaction with aminoethylcarbazol solution (Merck, Darmstadt, Germany), cells were counterstained with haematoxylin. Negative controls were conducted in all cases omitting primary antibody. For evaluation of immunocytochemical staining the total number of cells per 5 high-power fields (using x40 –HPF objective magnification) was determined. Cells were considered positive, when they demonstrated strong and exclusive labelling.

### Cell culture and reagents

Human embryonic kidney (HEK) cell line 293 T was maintained in Dulbecco’s modified Eagle’s medium (Invitrogen Life Technologies, Carlsbad, CA, USA). CRC cell line HT29 was cultivated in McCoy’s + GlutaMAX medium and CaCo2 cell line was maintained in MEM + GlutaMAX Medium. The medium contained 10% (v/v) Fetal Bovine Serum Gold (PAA Laboratories GmbH, Pasching, Austria) and 1% (v/v) Penicillin/Streptomycin (Invitrogen Life Technologies). All cell lines were cultured in a 5% CO_2_-humidified incubator (Nalge Nunc International, Rochester, NY, USA) at 37°C.

The Pre-miR miRNA Precursor of miR-518a-5p (Ambion Life Technologies, Carlsbad, CA, USA; Cat. No. 17100 ID: PM12865) and the negative control (Ambion Life Technologies, Cat. Nr. 17110) were purchased from Ambion Life Technologies.

### Calculations and statistical methods

CCR6 and luciferase expression profiles of the different groups are shown as mean and standard error of the mean (SEM). Comparison of luciferase activities between miR-518a-5p cotransfected groups with the luciferase vector constructs Luc No CCR6, Luc CCR6 and Luc CCR6 Mut1, 2 and 1 + 2 was performed with one way analysis of variance (ANOVA) with the Bonferroni-Post-hoc-Test. Where appropriate, the Student’s *t*-test was applied to test for group differences of continuous variables. All calculations were done with SPSS, version 19. The significance level was *p* < 0.05.

## Results

### Screening for microRNA target sites in the 3′UTR of CCR6

**Figure 1:**
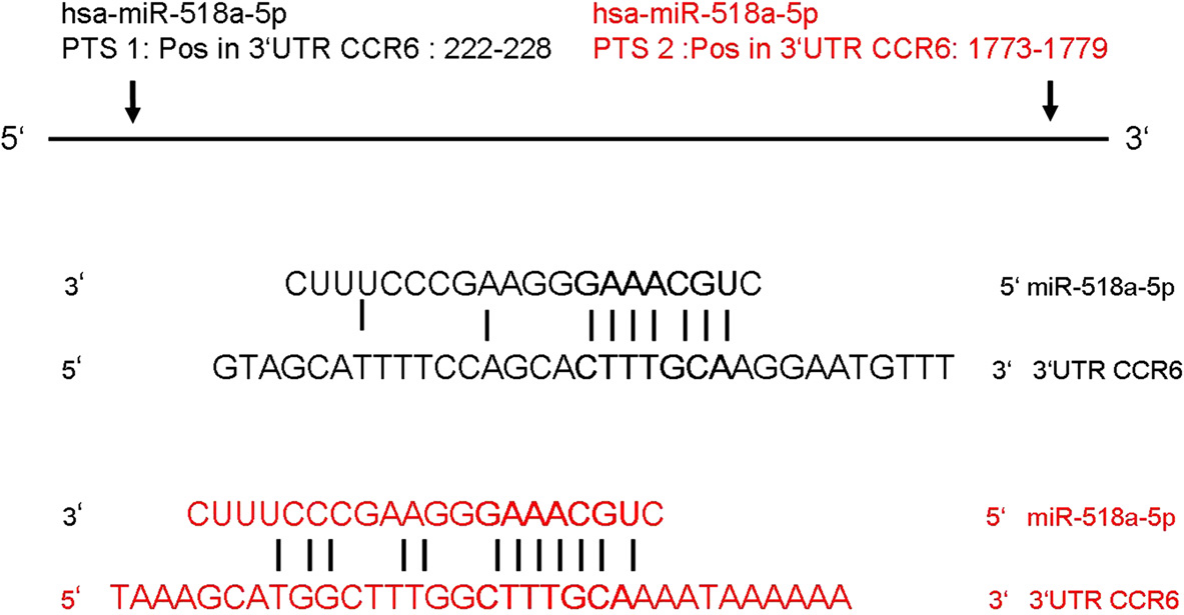
**Predicted miRNA target sites (PTSs) on the CCR6 3′UTR.** Hsa-miR-518a-5p was predicted by target prediction programs TargetScan Human 5.1, MiRDB and TargetMiner to align to positions 242–228 and 1773–1779 on the CCR6 3′UTR. Paired sequence alignment is marked by continuous lines.

To identify miRNAs that potentially interact with the 3′UTR of CCR6, various prediction software tools were applied. Only miRNAs that were identified by two or more out of the five queried target prediction programs were selected for experimental investigations. For miR-518a-5p, three of five target prediction programs under investigation, TargetScan Human 5.1, MiRDB and TargetMiner predicted a potential interaction with CCR6 as presented in Table 1 and Figure 1. As shown in Figure 1 the predicted alignment sequences of miR-518a-5p include positions 222–228 and 1773–1779 on the 3′UTR of CCR6.

### MicroRNA 518 functionally interacts with the 3′UTR of CCR6

**Figure 2:**
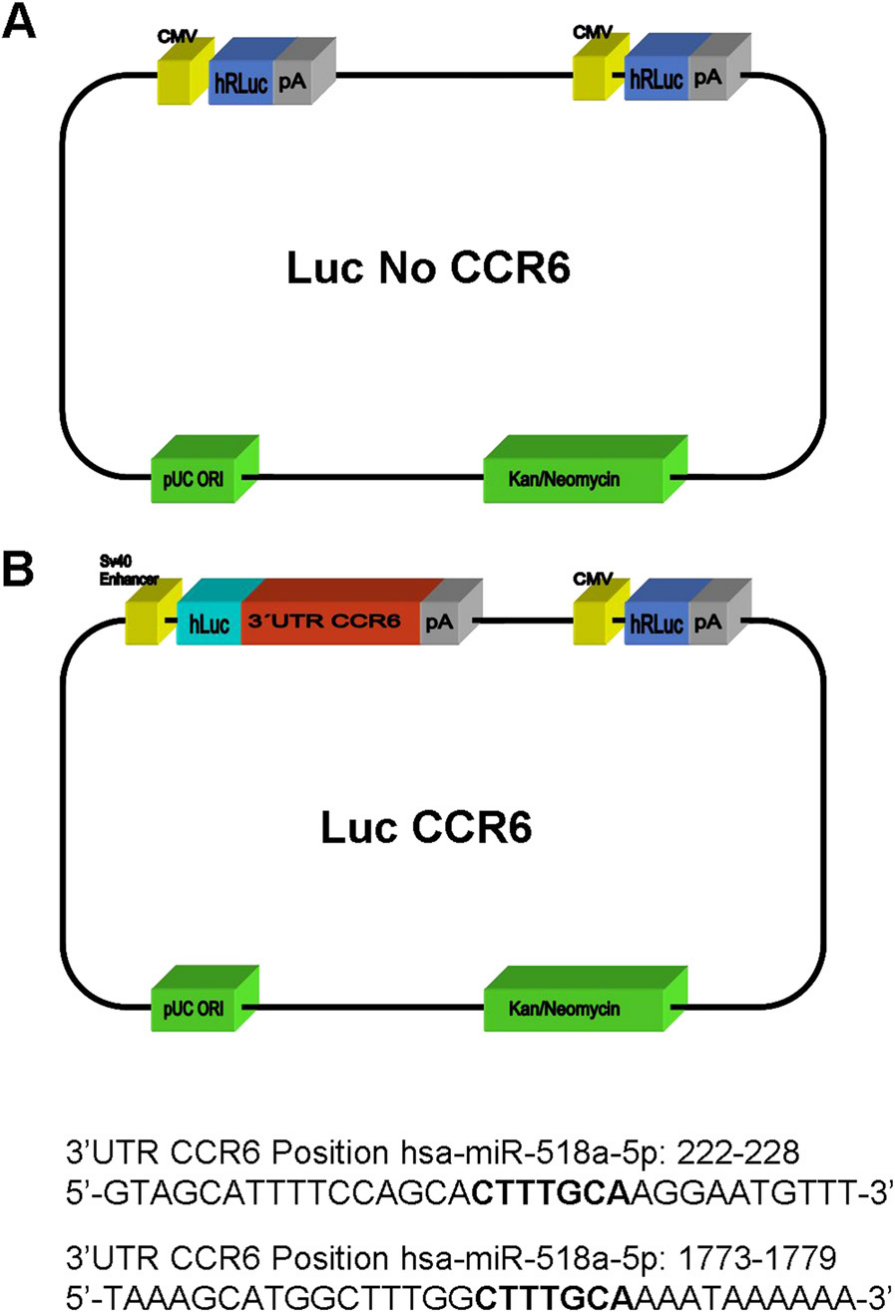
**Dual luciferase reporter assay system with and without 3′UTR of CCR6.** The luciferase reporter vectors used in the Dual Luciferase Reporter assay system contain either a control vector with no 3′UTR (Luc No CCR6) **(A)** or a vector with the 3′UTR of CCR6 downstream of the firefly luciferase gene (Luc CCR6) **(B)**. Predicted alignment sequence of Hsa-miR-518a-5p contains positions 222–228 and 1773–1779 on the 3′UTR of CCR6. Bold labels refer to the respective seed sequences of miR-518a-5p in the 3′UTR of CCR6.

**Figure 3:**
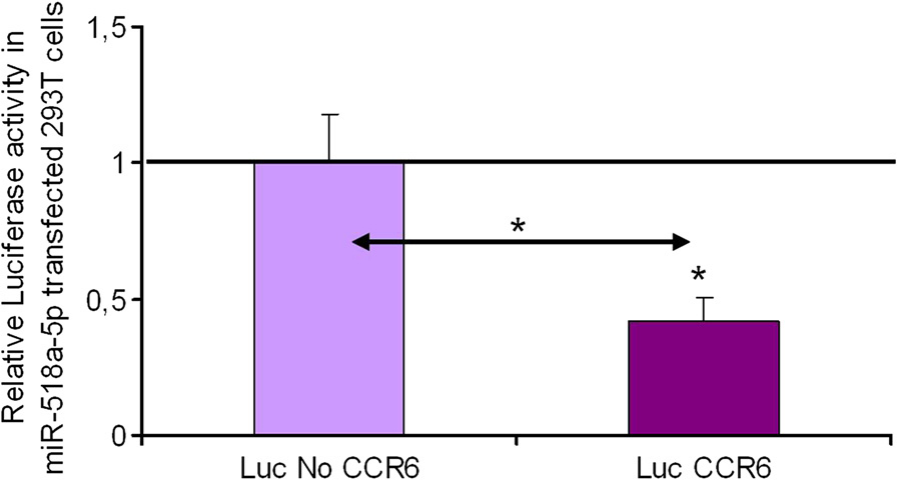
**Luciferase activity in miR transfected 293 T cells.** Luciferase activity in 293 T cells cotransfected with Luc CCR6 and Luc No CCR6 vectors, respectively, and miR-518a-5p relative to negative control (NK) miR mimic transfected cells. Luciferase activities are presented as mean +/- SEM (*n* = 10). Co-transfection with hsa-miR-518a-5p resulted in significant down-regulation of luciferase activity *(P < 0.05) with respect to Luc No CCR6 and NK miR mimic transfected cells. Fold decrease below 1 indicates luciferase down-regulation in Luc CCR6/hsa-miR-518a-5p cotransfected tissues related to NK miR mimic transfected cells.

Next, we examined whether miR-518a-5p potentially influences the regulation of CCR6 gene expression. Functional implications of miR-518a-5p with the 3′UTR of CCR6 were analyzed using the Dual Luciferase Reporter assay system. The luciferase reporter vectors contained either a control vector with no 3′UTR (Luc No CCR6-vector) or the 3′UTR of CCR6 downstream of the firefly luciferase gene (Luc CCR6 vector) as demonstrated in Figure 2A, B and C. Co-transfection of HEK cell line 293 T with the Luc CCR6 vector and miR-518a-5p precursors, respectively, resulted in a significant 60% down-regulation of luciferase activity (P < 0.05) as presented in Figure 3. Thus, miR-518a-5p regulates the expression of a luciferase construct which contains the 3′UTR of CCR6.

### Site-directed mutagenesis of the seed sequence verifies functional interaction of miR-518a-5p with CCR6

**Figure 4:**
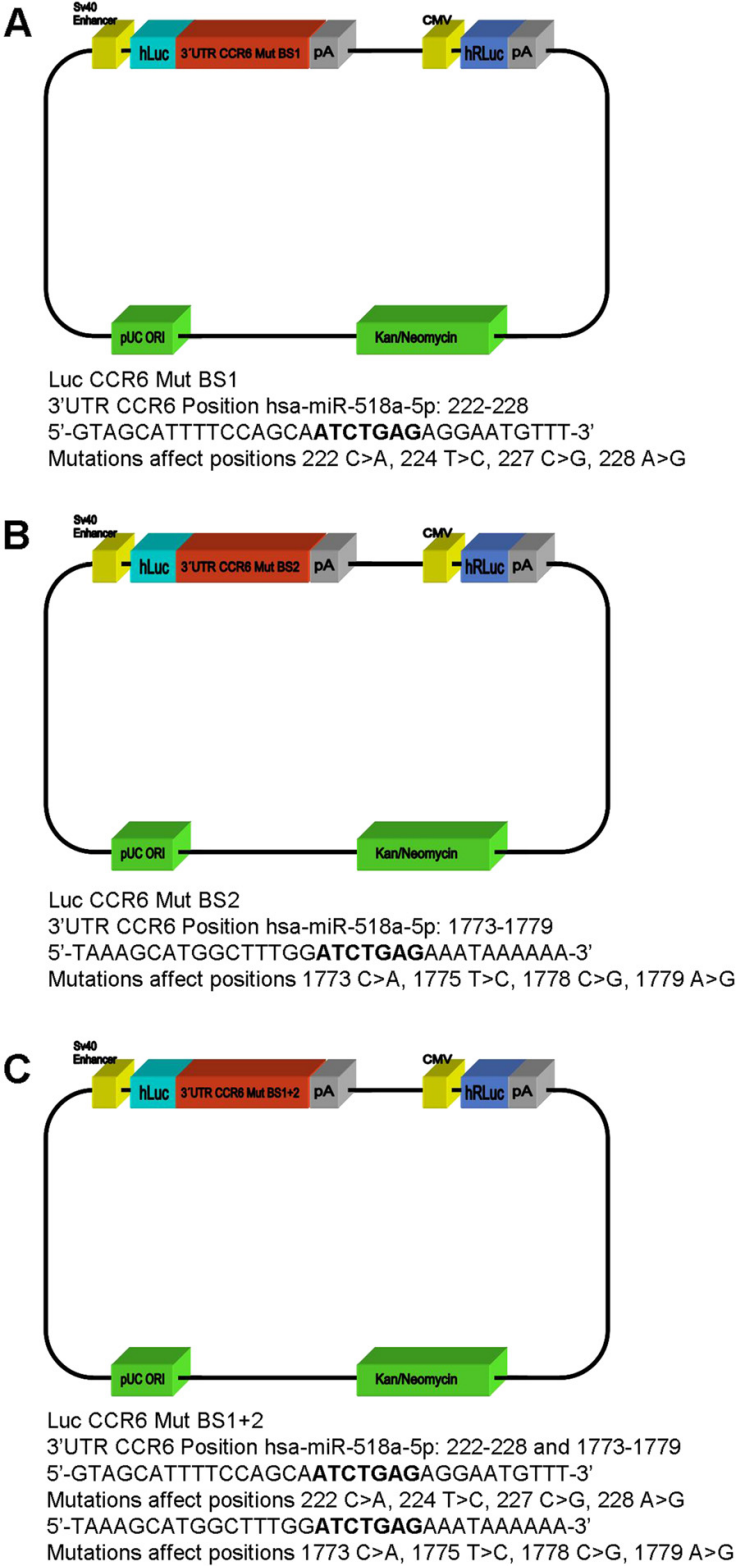
**Dual luciferase reporter assay system with mutated 3′UTR of CCR6.** Site-directed mutagenesis was introduced into the 2 seed sequences of hsa-miR-518a-5p in positions 222–228 and 1773–1779 in the 3′UTR of CCR6 resulting in three mutated luciferase expression vectors designated **(A)** Luc CCR6 Mut BS1, **(B)** Luc CCR6 Mut BS2 and **(C)** Luc CCR6 Mut BS1 + 2, respectively.

**Figure 5:**
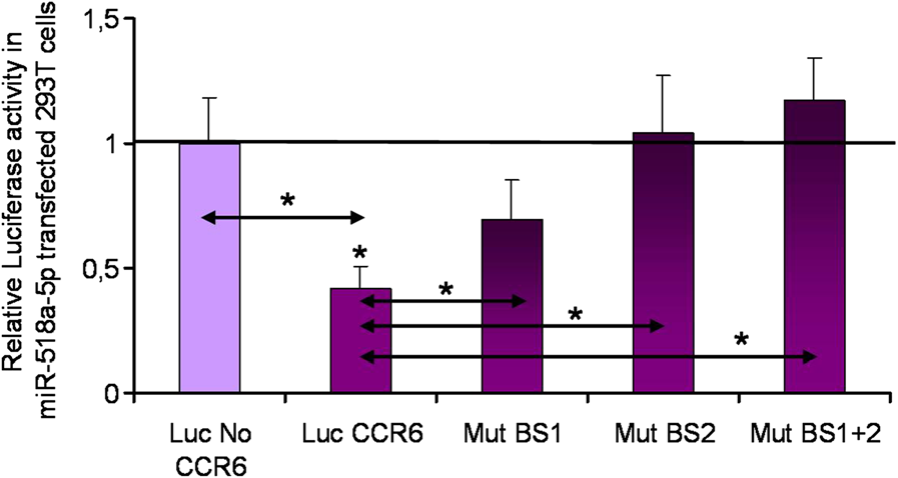
**Luciferase activity after site-directed mutagenesis in miR-518a-5p transfected 293 T cells.** Luciferase activity in 293 T cells cotransfected with Luc CCR6, Luc No CCR6 and Luc CCR6 Mut BS1, Luc CCR6 Mut BS2 and Luc CCR6 Mut BS1 + 2, respectively, and miR-518a-5p relative to NK miR mimic transfected cells. Luciferase activities are presented as mean +/- SEM (*n* = 10). Luciferase activity after Site Directed Mutagenesis of the 3′UTR of CCR6 in the miR-518a-5p Seed Sequences was significantly higher in all three mutated plasmids with respect to the Luc-CCR6 vector transfected cells *(P < 0.05). Fold decrease below 1 indicates luciferase down-regulation related to NK miR mimic transfected cells.

A site-directed mutagenesis spanning 4 bp was introduced into the two seed sequences of the 3′UTR of CCR6, respectively, resulting in three modified plasmids designated Luc CCR6 Mut BS1, Luc CCR6 Mut BS2 and Luc CCR6 Mut BS1 + 2, respectively, as presented in Figure 4. Mutations were confirmed by DNA sequencing of the mutated region. Subsequently, the mutated seed sequences were applied in a luciferase assay with miR-518a-5p mimics. We have demonstrated that luciferase activity observed with the Luc CCR6 Mut vectors is significantly up-regulated with respect to the Luc CCR6 vector in HEK 293 T cells as shown in Figure 5. In this respect, functional confirmation of the predicted target site for miR-518a-5p was achieved by site-directed mutagenesis of the seed sequences in the 3′UTR of CCR6 and subsequent application of the mutated seed sequences in a luciferase assay with miR-518a-5p mimics.

### miR-518a-5p down-regulates CCR6 expression in different CRC cell lines

**Figure 6:**
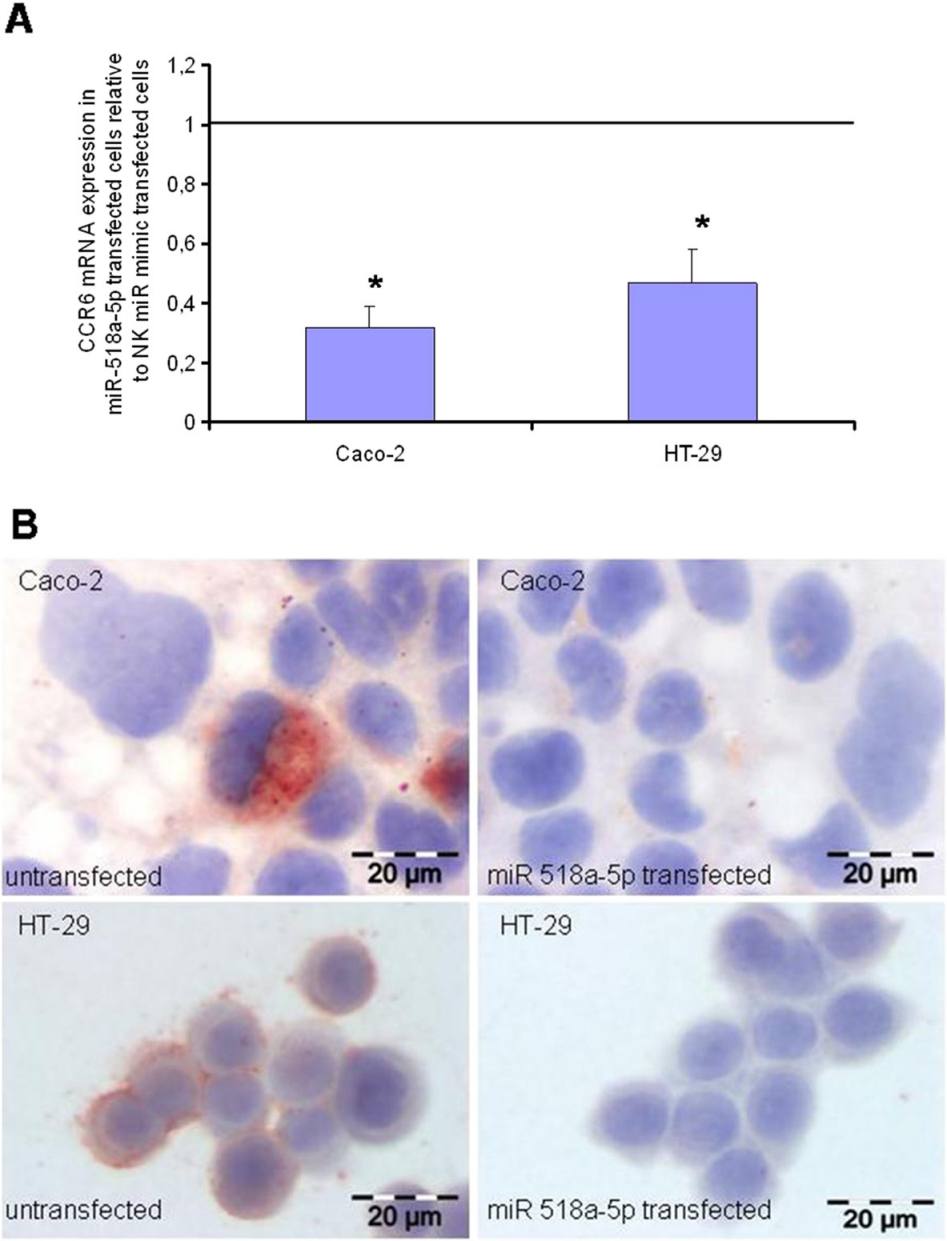
**CCR6 mRNA and protein expression in miR 518-5p transfected CRC cells. (A)** CCR6 mRNA expression as determined by Q-RT-PCR in CaCo2- and HT-29 cells 48 hours after transfection with hsa-miR-518a-5p. CCR6 Q-RT-PCR data are presented as mean +/- SEM (*n* = 15). Fold decrease below 1 indicates CCR6 mRNA down-regulation in CaCo2- and HT-29 cells relative to NK miR mimic transfected cells, *(P < 0.05). **(B)** Detection of CCR6 protein expression in representative cell culture slides of CaCo2- and HT-29 cells as assessed by immunocytochemical staining with CCR6-specific antibodies.

Following transfection of CRC cell lines with miR-518a-5p mimics, monitoring of CCR6 mRNA and protein expression was performed. For transfection of miRNA mimics two CRC cell lines with and without metastatic potential, HT-29 and Caco-2, respectively, were used. Gene expression of CCR6 was monitored after 48 hours after transfection with miR-518a-5p at the mRNA level and after 72 hours after transfection with miR-518a-5p at the protein level using Realtime PCR and immunocytochemistry, respectively. As presented in Figure 6A, CCR6 mRNA expression was significantly down-regulated in both cell lines under investigation, HT-29 and Caco-2, respectively (P < 0.05). These results were also reflected in CCR6 protein expression after immunocytochemical staining of both cell lines (Figure 6B). In Caco-2 CCR6 mRNA expression was significantly down-regulated by nearly 70% while in HT-29 CCR6 mRNA expression was significantly down-regulated by approximately 50%.

## Discussion

In this study we investigated functional interactions between miR-518a-5p and chemokine receptor CCR6. Hereby, we have provided evidence that miR-518a-5p regulates the expression of a luciferase construct containing the 3′UTR of CCR6. Moreover, we have demonstrated that in two CRC cell lines, Caco-2 and HT-29, respectively, artificial miR-518a-5p over-expression by transfection with miR-518a-5p mimics leads to significantly decreased expression of CCR6 both at the mRNA and protein level. Consequently, it appears that miR-518a-5p regulates CCR6 expression by a regulatory element present in the 3′UTR of CCR6. It may be deduced that miR-518a-5p functionally interacts with the 3′UTR of CCR6 and down-regulates CCR6 expression in CRC cell lines.

Functional confirmation of the predicted target site for miR-518a-5p was achieved by site-directed mutagenesis of the seed sequences in the 3′UTR of CCR6 and subsequent application of the mutated seed sequences in a luciferase assay with miR-518a-5p mimics. While co-transfection of HEK cell line 293 T with the Luc CCR6 vector and miR-518a-5p precursors, respectively, resulted in a significant 60% down-regulation of luciferase activity, transfection with Luc CCR6 Mut BS2 and Luc CCR6 Mut BS1 + 2, respectively, totally repressed the inhibitory effect of miR-518a-5p on luciferase expression and restored luciferase expression back to 100%. Therefore, mutation of one of the predicted target site alone and in combination with the other predicted target site entirely inhibit the effect of the miRNA. Therefore, site-directed mutagenesis of the seed sequences verified the functional interaction of miR-518a-5p with CCR6. In contrast, transfection with Luc CCR6 Mut BS1 alone did not entirely repress the inhibitory effect of miR-518a-5p on luciferase expression. If there is more than one target site for one miRNA in the 3′UTR of a target gene, the mutation of one target site may not be able to entirely inhibit the effect of the miRNA, whereas mutating both sites often totally inhibits miRNA mediated expression inhibition [26]. This mechanism may impact the 3′UTR of CCR6 with respect to miR-518a-5p. However, luciferase activity observed with the Luc CCR6 Mut BS1 alone was still demonstrated to be significantly up-regulated with respect to the intact Luc CCR6 vector without a mutation in the 3′UTR of CCR6. Comparing luciferase activity observed with Luc CCR6 Mut BS1 to the luciferase activity observed with the Luc No CCR6 vector, we did not observe any significant difference. Although luciferase activity was not entirely restored after transfection with the vector carrying only a mutation in the first seed sequence, the inhibitory effect of miR-518a-5p on luciferase expression was still significantly counteracted by the mutation in the first seed sequence. Therefore, our results reflect a comprehensible image of the successive counteracting effects of the different mutational steps in the 3′UTR of CCR6 on the luciferase activity after transfection with the different mutated vectors. Our results also demonstrate that a site-directed mutagenesis spanning 4 bp introduced into the respective seed sequences is effectually disrupting the interaction of miR-518a-5p with the 3′UTR of CCR6. To date, various studies have shown that mutating 3–5 nucleotides is sufficient to disrupt miRNA/mRNA interaction [27]. Mutating more nucleotides or deleting the binding site may achhieve a total repression of miRNA interaction. Such an effect was delineated for the interaction of mir-27b with PPAR mRNA [28].

Initially, we also investigated on the basis of alignment studies if there were other miR-518a-5p binding sites in the 3′UTR or in the coding DNA sequence of CCR6. In this respect, we detected several other imperfect complementarity interactions which may repress the protein translation to some extent. However, the software programmes have chosen the seed sequence on the basis of calculations that consider among other factors the melting temperature and the 3D model of the molecule thus being able to predict if binding is on principle sterically feasible. Consequently, there may be imperfect complementarity interactions based on our alignment studies, but the seed sequence calculated by the software tools may be the only one that sterically fits the 3D model and therefore allows binding in vivo causing the effect of downregulation of luciferase activity. This was confirmed by mutating the seed sequence as described above, which totally repressed the inhibitory effect of miR-518a-5p on luciferase expression and restored luciferase expression back to 100% when transfected with Luc CCR6 Mut BS2 and Luc CCR6 Mut BS1 + 2, respectively. If other imperfect binding sites were causing the effect of down-regulation of luciferase activity, mutation of the seed sequence alone could not have reversed the effect. In this way functional confirmation of the predicted target site for miR-518a-5p was unambigiously shown.

Screening for microRNA target sites in the 3′UTR of CCR6 identified several miRNAs that potentially interact with the 3′UTR of CCR6. In this context, we have found three other miRNAs predicted by the target prediction programmes to bind to the 3′UTR of CCR6. As miR-518a-5p was predicted by three of the five prediction programmes, we have started our investigations with this miRNA. However, at this stage we report a functional interaction between miR-518a-5p and its identified chemokine receptor target CCR6 in CRC cell lines. Further investigations will show if such functional interactions are permitted on the cellular level which would require an inverse correlation of expression and also co-expression of miR-518a-5p and CCR6 by the same cell. In this respect, the other three miRNAs predicted by the software prediction programmes may also turn out to be interesting candidates for in vitro and in vivo interactions with CCR6.

Following luciferase assays we demonstrated that miR-518a-5p also down-regulates CCR6 expression in different CRC cell lines. For CRC cell lines we have chosen one cell line with metastatic potential, HT-29, and another cell line without metastatic potential, Caco-2, respectively. 48 hours after transfection with miR-518a-5p mimics CCR6 mRNA expression was monitored and shown to be significantly down-regulated in both cell lines which was also demonstrated for CCR6 protein expression 72 hours after transfection with miR-518a-5p mimics by immunocytochemical staining of both cell lines. In this way, we have shown that independently of the metastatic potential of a CRC cell line artificial miR-518a-5p over-expression by transfection of miR-518a-5p mimics leads to significantly decreased expression of CCR6 on the mRNA and protein level. The latter may have been achieved by inhibition of translation or mRNA degradation which may depend on the degree of complementarity between the microRNA sequence and the target sequence in the 3′UTR region [1]. High complementarity leads to degradation of mRNA while lower complementarity leads to translation repression [14,29].

As we have shown, expression of CCR6 and its corresponding ligand CCL20 is significantly dysregulated in CRC and colorectal liver metastasis when compared with normal mucosa [15,16,30]. Consequently, the CCR6/CCL20 system may be involved in the molecular mechanisms controlling CRC pathology. In this context, recent data support an involvement of the CCR6/CCL20 system in CRC pathology. One study reported that tumor-associated macrophages recruit CCR6+ regulatory T cells to tumor mass and promote its development via enhancing the production of CCL20 in a CRC mouse model [31]. Based on a multivariable analysis another study suggested that preoperative serum carcinoembryonic antigen level of CCR6 was an independent factor associated with distant metastasis [32]. Consequently, it was concluded that the expression of CCR6 in CRC could predict metachronous distant metastasis.

Recently, we have demonstrated that miR-21 functionally interacts with 3′UTR of CCL20 and verified this interaction in CRC cells [22]. To date, there are no data investigating putative interactions of the sole CCL20 receptor CCR6 with candidate miRNAs. However, recent studies allocate a role in CRC pathogenesis to various miRNAs. MiRNAs are associated with the development and progression of CRC, partly by regulating the expression of oncogenes and tumour suppressors and partly by functioning as oncogenes or tumour suppressors themselves [33,34] Accordingly, in CRC the expression of various miRNAs has been demonstrated to be aberrantly expressed, mainly down-regulated [20,21]. Artificial dysregulation of certain miRNAs will trigger tumorigenesis or apoptosis and will influence CRC prognosis. As a result, overexpression of miR-21 is associated with worse prognosis and poorer response to chemotherapeutics in CRC. Recently, miR-21 was demonstrated to post-transcriptionally down-regulate tumor suppressor Pdcd4 and overexpression of miR-21 stimulated tumor cells to invade, intravasate and metastasise more aggressively when implanted into CRC mouse models [35]. Little is known for miR-518a-5p with respect to a role in cancer. To date, miR-518a-5p is speculated as a putative candidate for an involvement in the development of cervical carcinoma as it was found to be differentially regulated in high-grade CIN specimens and cervical squamous cell carcinoma with respect to normal cervical epithelium [36].

Increasing evidence indicates that the global transcriptional down-regulation of miRNAs in CRC is caused by epigenetic processes [37]. In this respect, miR-137 and miR-342 both act as tumour suppressors and are frequently silenced by promoter hypermethylation in early stages of CRC [37,38]. In addition, miR-137 was shown to target cdc42 expression, inducing cell cycle g1 arrest and inhibiting invasion in CRC cells [39]. Also miR-185 was shown to target cdc42 and RhoA expression thus inhibiting the proliferation potential of CRC cells [40] while miR-135 affects the Wnt signalling pathway by downregulating the tumour suppressor gene Adenomatous Polyposis Coli (APC) [41]. Another potential tumour-suppressive miRNA in CRC development is miR-143, which might regulate DNA methylation by targeting DNA methyltransferase 3A (DNMT3A) [42]. Although the role of miRNAs in the development of CRC metastasis has been investigated using in vitro assays, mouse models and tissue-based experiments in CRC patients, the amount of data is still limited. However, cells with a permanent inactivation of tumour suppressive factors have a selective advantage to metastasize. Hence, miR-34a, a member of the miR-34 family (consisting of miR-34a, miR-34b, and miR-34c) was identified as a direct downstream transcriptional target of the multifunctional tumour suppressor gene TP53 [43]. Many miR-34a-responsive genes regulate cell cycle progression, cellular proliferation (E2F) or apoptosis (BCL2). Therefore, transfection of CRC cells with miR-34a induces senescence and apoptotic cell death [43,44]. However, if CRC cells show the presence of TP53 mutations [45] a significant number of primary tumours demonstrate decreased miR-34a expression [44]. and therefore have a higher ability to metastasize. Alternatively, epigenetic inactivation of the miR-34 family by promoter hypermethylation is observed in many CRC cell lines [46,47] and primary CRCs [48,49].

Based on previous results that demonstrated aberrant expression of chemokine receptor CCR6 in CRC tissues, we hypothesized that CCR6 may be aberrantly regulated by miRNAs in CRC. To test this hypothesis, we aimed to identify miRNAs that regulate CCR6 expression in CRC cells. In summary, our results indicate a direct functional interaction of miR-518a-5p with the chemokine target gene CCR6 in CRC cells. Further, we have demonstrated that miR-518a-5p down-regulates CCR6 expression in miR-518a-5p transfected CRC cell lines. Hence, we have shown that miR-518a-5p regulates CCR6 expression in CRC cells which may be a general regulatory mechanism involved in the development and progression of CRC.

## Conclusions

In conclusion, our results provide evidence that miR-518a-5p functionally interacts with the 3′UTR of CCR6. It is further documented that transfection of CRC cells with miR-518a-5p leads to significant CCR6 down-regulation on the mRNA and protein level. Consequently, our findings may aid in the understanding of miRNA gene regulation with respect to chemokines and their interaction in CRC cells emphasizing the importance of further studies of the regulative mechanism underlying the miR-518a-5p/CCR6 interaction.

## Competing interests

The authors declare that they have no competing interests and there are NO relationships that could be construed as resulting in an actual, potential, or perceived conflict of interest with regard to the manuscript submitted for review.

## Authors’ contributions

CR: Study concept and design, Drafting of the manuscript, Analysis and interpretation of data. BK: Acquisition of data, Analysis and interpretation of data. VOF: Technical support, interpretation of data. KK: Technical support, interpretation of data. PG: Critical revision of the manuscript for important intellectual content. MW: Technical support. FG: Technical support. SG: Statistical analysis. MKS: Material support. All authors read and approved the final manuscript.
